# 

**Published:** 1890-09

**Authors:** 


Entered at the Post Office at Austin, Texas, as Second-Class Mail Matter.

Vol. VI. SEPTEMBER, 1890. No. 2.

### DANIEL'S TEXAS MEDICAL JOURNAL

Subscription, $2.00 a Year, in Advance. — Single Copies, Twenty-five Cents.

F. E. DANIEL, M. D., Editor and Publisher, AUSTIN, TEXAS.

Eugene Von Boeckmann, Steam Book and Job Printer, Austin, Texas.

